# On the secrecy performance of transmit-receive diversity and spatial multiplexing systems

**DOI:** 10.7717/peerj-cs.186

**Published:** 2019-04-22

**Authors:** Kiattisak Maichalernnukul

**Affiliations:** College of Digital Innovation and Information Technology, Rangsit University, Pathum Thani, Thailand

**Keywords:** Physical-layer security, Secrecy outage probability, Transmit-receive diversity, Multiple-Input Multiple-Output, Spatial multiplexing

## Abstract

Emerging from the information-theoretic characterization of secrecy, physical-layer security exploits the physical properties of the wireless channel for security purpose. In recent years, a great deal of attention has been paid to investigating the physical-layer security issues in multiple-input multiple-output (MIMO) wireless communications. This paper analyzes the secrecy performance of transmit-receive diversity system and spatial multiplexing systems with zero-forcing equalization and minimum mean-square-error equalization. Specifically, exact and asymptotic closed-form expressions are derived for the secrecy outage probability of such MIMO systems in a Rayleigh fading environment, and the corresponding secrecy diversity orders and secrecy array gains are determined. Numerical results are presented to corroborate the analytical results and to examine the impact of various system parameters, including the numbers of antennas at the transmitter, the legitimate receiver, and the eavesdropper. These contributions bring about valuable insights into the physical-layer security in MIMO wireless systems.

## Introduction

Wireless communication systems are intrinsically prone to eavesdropping because of the open nature of the wireless medium. In this context, physical-layer security arising from the information-theoretic analysis of secrecy has attracted a lot of interest so far. This approach indeed takes advantage of the physical characteristics of the radio channel to support secure communications. Groundbreaking works on physical-layer security (Wyner, 1975; Csiszár & Körner, 1978; Leung-Yan-Cheong & Hellman, 1978; Bloch et al., 2008) focused on a basic wiretap channel, where the transmitter, the legitimate receiver, and the eavesdropper possess a single antenna, and established the so-called secrecy capacity. One of their common remarks was that to have a positive secrecy capacity, the channel quality of the transmitter–receiver link has to be better than that of the transmitter-eavesdropper link.

Stimulated by advances in multiple-antenna technology for wireless communications, the physical-layer security issues in multiple-input multiple-output (MIMO) wiretap channels^1^ 1In our context, a MIMO wiretap channel implies that there are multiple antennas at the transmitter, the legitimate receiver, and the eavesdropper. This is generally known as co-located MIMO. For a discussion on its alternative, called distributed or cooperative MIMO, readers are referred to (Dong et al., 2010; He, Man & Wang, 2011; Zou, Wang & Shen, 2013; Wang et al., 2016a).have been recently explored in the literature (Goel & Negi, 2008; Khisti & Wornell, 2010; Oggier & Hassibi, 2011; Mukherjee & Swindlehurst, 2011; Yang et al., 2013; Ferdinand, Da Costa & Latva-aho, 2013; Lin, Tsai & Lin, 2014; Wang, Wang & Ng, 2015; Schaefer & Loyka, 2015; Wang et al., 2016b; Maichalernnukul, 2018). A brief overview of these works is provided in the following subsection.

### Related works

In Khisti & Wornell (2010), a closed-form expression for the secrecy capacity of the Gaussian MIMO wiretap channel was derived from solving a minimax problem. Meanwhile, the problem of computing the perfect secrecy capacity of such a channel was analytically investigated in Oggier & Hassibi (2011). By relaxing the assumption of perfect channel state information (CSI) used in Khisti & Wornell (2010), Oggier & Hassibi (2011), Schaefer & Loyka (2015) studied the secrecy capacity of the compound Gaussian MIMO wiretap channel. In Mukherjee & Swindlehurst (2011), a few beamforming schemes were proposed to improve the secrecy capacity of the Gaussian MIMO wiretap channel in the presence of CSI errors. With the objective of achieving perfect secrecy at the physical layer, MIMO precoding and postcoding designs using the signal-to-noise ratio (SNR) criterion were presented in Lin, Tsai & Lin (2014).

In all aforementioned works, the channel was assumed to be fixed over the whole transmission time. More precisely, the channel gains for the Gaussian MIMO wiretap channel are constant. This is rarely practical for the wireless medium as multipath propagation normally makes transmission conditions vary with time (Poor & Schaefer, 2017). Such variation is called fading. In (Yang et al., 2013; Ferdinand, Da Costa & Latva-aho, 2013; Maichalernnukul, 2018), the secrecy capacity of the fading MIMO wiretap channel^2^ 2For this kind of channel, the channel gains are allowed to change from channel use to channel use (Poor & Schaefer, 2017).was characterized. Specifically, Yang et al. (2013) focused on the physical-layer security enhancement through transmit antenna selection in a flat-fading MIMO channel, and characterized the corresponding performance in terms of the secrecy outage probability and the probability of non-zero secrecy capacity. In the meantime, Ferdinand, Da Costa & Latva-aho (2013) analyzed the secrecy outage probability of orthogonal space–time block code (OSTBC) MIMO systems when the transmitter–receiver and transmitter-eavesdropper links experience different kinds of fading. In contrast to space–time coding (which is based on transmit diversity), transmit beamforming and receive combining (which is based on transmit-receive diversity) achieve additional array gain (Tse & Viswanath, 2005). Besides, Goel & Negi (2008) showed that multiple transmit antennas can be deployed to generate artificial noise, such that only the transmitter-eavesdropper link is degraded. This idea enables secret communication (Csiszár & Körner, 1978) and has been extended to more practical MIMO scenarios, e.g., frequency-division duplex systems (Wang, Wang & Ng, 2015) and heterogeneous cellular networks (Wang et al., 2016b).

More recently, in Maichalernnukul (2018), the average secrecy capacity of transmit-receive diversity systems in the fading MIMO wiretap channel and its upper bound were derived in closed form. Nevertheless, the corresponding secrecy outage probability has not been investigated yet. There are two reasons why we should study this performance. First, the closed-form results of Maichalernnukul (2018) are complicated, and from these results, it is not clear how the system parameters (e.g., the numbers of antennas at the transmitter, the legitimate receiver, and the eavesdropper) affect the secrecy performance. In fact, quantifying the secrecy outage probability at high SNR in terms of two parameters, namely secrecy diversity order and secrecy array gain, can provide insights into this effect (Yang et al., 2013). Second, it was shown in Bashar, Ding & Li (2011) that although transmit beamforming in the transmit-receive diversity systems maximizes the achievable capacity of the main channel (i.e., that for the transmitter–receiver link), they still have secrecy outages at an arbitrary target secrecy rate. The first objective of our work is to present the exact and asymptotic (high-SNR) analysis of the secrecy outage probability of these systems.

It is well known that the multiple antennas of MIMO systems can be exploited to obtain spatial multiplexing, i.e., transmission of independent data streams in parallel (Tse & Viswanath, 2005). This leads to an increase in the data rate. While several key performance metrics of spatial multiplexing MIMO systems, e.g., error probability, outage and ergodic capacity, have been extensively studied in the literature (Chen & Wang, 2007; Smith, 2007; Ordóñez et al., 2007; Kumar, Caire & Moustakas, 2009; Jiang, Varanasi & Li, 2011), little is known about the secrecy performance of these systems in the fading MIMO wiretap channel. The second objective of our work is to fill this knowledge gap by providing a relevant secrecy outage probability characterization.

### Contributions

The main contributions of this work are summarized as follows:

• We derive exact and asymptotic closed-form expressions for the secrecy outage probability of a transmit-receive diversity system in the fading MIMO wiretap channel. We also do the same for the secrecy outage probability of spatial multiplexing systems with linear equalization, especially zero-forcing (ZF) and minimum mean-square-error (MMSE).^3^ 3The rationale for using these “classical” detection techniques for the spatial multiplexing MIMO systems is twofold. First, the ZF and MMSE detectors are the basic building blocks of advanced MIMO communication architectures (e.g., layered space–time architectures (Foschini, 1996; Seethaler, Artés & Hlawatsch, 2004) and joint transmit-receive equalizers (Palomar & Lagunas, 2003; Jiang, Li & Hager, 2005)), and have been extensively addressed in the MIMO literature (Jankiraman, 2004; Biglieri et al., 2007; Heath Jr & Lozano, 2018). Second, they have low computational complexity compared to the (optimum) maximum likelihood (ML) detector, and their performance can be very close to the ML performance for a well-conditioned MIMO channel, i.e., its condition number is near to unity (see Seethaler, Artés & Hlawatsch (2005) for more details).It is shown that all exact secrecy outage results simplify to the well-known result (Bloch et al., 2008, Equation (9)) for the case where the transmitter, the legitimate receiver, and the eavesdropper have a single antenna.
• We determine the secrecy diversity order and secrecy array gain that the above systems achieve, and discuss the impact of the numbers of antennas at the transmitter, the legitimate receiver, and the eavesdropper, denoted as *M*_t_, *M*_r_, and *M*_e_, respectively, on the system secrecy and complexity. Through numerical results, it is verified that the transmit-receive diversity system attains a secrecy diversity order of *M*_t_*M*_r_, while the spatial multiplexing systems with ZF equalization and MMSE equalization yield the same secrecy diversity order of *M*_r_ − *M*_t_ + 1. All of these secrecy diversity orders turn out to be independent of *M*_e_.

### Notation and organization

Throughout this paper, we write a function *g*(*x*) of variable *x* as *o*(*x*) if ${\lim }_{x\rightarrow 0} \frac{g(x)}{x} =0$, and denote $ \left( {\cdot \atop \cdot } \right) $ as the multinomial coefficient, E[⋅] as the expectation operator, $ \frac{\text{d}}{\text{d}x} (\cdot )$ as the first derivative operator with respect to variable *x*, ∥⋅ ∥ as the Euclidean norm of a vector, and **I**_*N*_ as the identity matrix of size *N* × *N*. Moreover, det(⋅), (⋅)^T^, (⋅)^†^, (⋅)^−1^, and [⋅]_*ij*_ denote the determinant, transpose, conjugate transpose, inverse, and (*i*, *j*)-th element of a matrix, respectively, and ϒ(⋅, ⋅) and Γ(⋅, ⋅) are the lower and upper incomplete gamma functions defined in (Gradshteyn & Ryzhik, 2000, Equation (8.350.1)) and (Gradshteyn & Ryzhik, 2000, Equation (8.350.2)), respectively. We also denote $\mathcal{CN}(\mathbf{0},\mathbf{K})$ as a zero-mean circularly-symmetric complex Gaussian distribution with covariance **K** (Gallager, 2008, Section 7.8.1), and ${\mathcal{L}}_{\text{max}}\{&sdot; \}$ and $\mathcal{P}\{&sdot; \}$ as the largest eigenvalue of a square matrix and the associated eigenvector, respectively.

The layout of the paper is as follows. ‘System Model’ describes the system model of interest. ‘Exact Secrecy Outage Probability’ and ‘Asymptotic Secrecy Outage Probability’ present exact and asymptotic analysis of the corresponding secrecy outage probability, respectively. ‘Numerical Results’ provides the numerical results of theoretical analysis and simulations, followed by the conclusion given in ‘Conclusion’.

## System Model

In this section, we consider transmit-receive diversity and spatial multiplexing systems where the transmitter, the legitimate receiver, and the passive eavesdropper are equipped with *M*_t_, *M*_r_, and *M*_e_ antennas, respectively. The instantaneous secrecy capacity of these systems is given by (Bloch et al., 2008, Lemma 1)

(1) \begin{eqnarray*}{C}_{\text{s}}= \left\{ \right. \begin{array}{@{}lr@{}} \displaystyle {\log \nolimits }_{2} \left( 1+{\gamma }_{\text{r}} \right) -{\log \nolimits }_{2} \left( 1+{\gamma }_{\text{e}} \right) ,&\displaystyle \text{if}{\gamma }_{\text{r}}\gt {\gamma }_{\text{e}}\\ \displaystyle 0,&\displaystyle \text{if}{\gamma }_{\text{r}}\leq {\gamma }_{\text{e}}\\ \displaystyle \end{array}\end{eqnarray*}

where *γ*_r_ and *γ*_e_ are the instantaneous received SNRs at the receiver and the eavesdropper, respectively.

### Transmit-receive diversity system

For the transmit-receive diversity system, the received signal vector at the legitimate receiver, **y**_r_ ∈ ℂ^*M*_r_×1^, and that at the passive eavesdropper, **y**_e_ ∈ ℂ^*M*_e_×1^, depend on the transmitted symbol *s* ∈ ℂ (with E[|*s*|^2^] = *P*) according to

(2) \begin{eqnarray*}{\mathbf{y}}_{\text{r}}={\mathbf{H}}_{\text{r}}{\mathbf{w}}_{\text{t}}s+{\mathbf{n}}_{\text{r}}\end{eqnarray*}

and

(3) \begin{eqnarray*}{\mathbf{y}}_{\text{e}}={\mathbf{H}}_{\text{e}}{\mathbf{w}}_{\text{t}}s+{\mathbf{n}}_{\text{e}}\end{eqnarray*}

respectively, where **w**_t_ ∈ ℂ^*M*_t_×1^ is the transmit weight (beamforming) vector, and **n**_r_ and **n**_e_ are independent circularly-symmetric complex-valued Gaussian noises: ${\mathbf{n}}_{\text{r}}\sim \mathcal{CN}(\mathbf{0},{\sigma }_{\text{r}}^{2}{\mathbf{I}}_{{M}_{\text{r}}})$ and ${\mathbf{n}}_{\text{e}}\sim \mathcal{CN}(\mathbf{0},{\sigma }_{\text{e}}^{2}{\mathbf{I}}_{{M}_{\text{e}}})$. We focus on a Rayleigh-fading wiretap channel, meaning that the channel matrices **H**_r_ and **H**_e_ have independent identically-distributed $\mathcal{CN}(0,1)$ entries. In addition, we assume that the three terminals know **H**_r_, but **H**_e_ is available only at the eavesdropper.^4^ 4This assumption holds, for example, if the receiver and eavesdropper are able to perfectly estimate **H**_r_ and **H**_e_, respectively, and the receiver sends **H**_r_ to the transmitter through a noiseless broadcast channel, which can be heard by the eavesdropper (Goel & Negi, 2008).

The receiver estimates the symbol *s* by applying the receive weight (combining) vector **z**_r_ to the received signal vector **y**_r_:

\begin{eqnarray*}{\mathbf{z}}_{\text{r}}^{\dagger }{\mathbf{y}}_{\text{r}}={\mathbf{z}}_{\text{r}}^{\dagger }{\mathbf{H}}_{\text{r}}{\mathbf{w}}_{\text{t}}s+{\mathbf{z}}_{\text{r}}^{\dagger }{\mathbf{n}}_{\text{r}}. \end{eqnarray*}

The optimal choices of **w**_t_ and **z**_r_ in the sense of maximizing the SNR of this estimate (i.e., the instantaneous received SNR) are given by Dighe, Mallik & Jamuar (2003)

\begin{eqnarray*}{\mathbf{w}}_{\text{t}}= \frac{{\mathbf{H}}_{\text{r}}^{\dagger }{\mathbf{z}}_{\text{r}}}{\parallel {\mathbf{H}}_{\text{r}}^{\dagger }{\mathbf{z}}_{\text{r}}\parallel } \end{eqnarray*}

and

\begin{eqnarray*}{\mathbf{z}}_{\text{r}}=\mathcal{P}\{{\mathbf{H}}_{\text{r}}{\mathbf{H}}_{\text{r}}^{\dagger }\} \end{eqnarray*}

respectively, and the resultant SNR is

(4) \begin{eqnarray*}{\gamma }_{\text{r,TR}}={\bar {\gamma }}_{\text{r}}{\mathcal{L}}_{\text{max}}\{{\mathbf{H}}_{\text{r}}{\mathbf{H}}_{\text{r}}^{\dagger }\}\end{eqnarray*}

where ${\bar {\gamma }}_{\text{r}}= \frac{P}{{\sigma }_{\text{r}}^{2}} $ is the average SNR at the receiver. The subscript TR refers to the transmit-receive diversity system, and is sometimes used to avoid confusion between this system and the spatial multiplexing system. Let $\lambda ={\mathcal{L}}_{\text{max}}\{{\mathbf{H}}_{\text{r}}{\mathbf{H}}_{\text{r}}^{\dagger }\}$, *L* = min(*M*_t_, *M*_r_), and *K* = max(*M*_t_, *M*_r_). The cumulative distribution function (CDF) of *λ* is given by Dighe, Mallik & Jamuar (2003)

(5) \begin{eqnarray*}{F}_{\lambda }(x)= \frac{\det \nolimits (\mathbf{S}(x))}{ \left[ \prod _{p=1}^{L}(K-p){!}(L-p){!} \right] } \end{eqnarray*}

where **S**(*x*) is the *L* × *L* Hankel matrix with

\begin{eqnarray*}[\mathbf{S}(x)]_{ij}=\Upsilon ( \left\vert {M}_{\text{t}}-{M}_{\text{r}} \right\vert +i+j-1,x). \end{eqnarray*}

By careful inspection of the entries of **S**(*x*), this CDF can be rewritten as

(6) \begin{eqnarray*}{F}_{\lambda }(x)=\sum _{m=1}^{L}\sum _{n= \left\vert {M}_{\text{t}}-{M}_{\text{r}} \right\vert }^{({M}_{\text{t}}+{M}_{\text{r}}-2m)m} \frac{{a}_{m,n}}{n{!}} \Upsilon \left( n+1,mx \right) \end{eqnarray*}

where ${a}_{m,n}= \frac{{c}_{m,n}n{!}}{{m}^{n+1} \left[ {\mathop{\prod }\nolimits }_{p=1}^{L}(K-p){!}(L-p){!} \right] } $ and *c*_*m*,*n*_ is the coefficient computed by using curve fitting on the plot of $ \frac{\text{d}}{\text{d}x} \det (\mathbf{S}(x))$ (Dighe, Mallik & Jamuar, 2003). Using Eq. (6) and (Papoulis & Pillai, 2002, Example 5-1), the CDF of *γ*_r,TR_ in Eq. (4) is given by

(7) \begin{eqnarray*}{F}_{{\gamma }_{\text{r,TR}}}(x)=\sum _{m=1}^{L}\sum _{n= \left\vert {M}_{\text{t}}-{M}_{\text{r}} \right\vert }^{({M}_{\text{t}}+{M}_{\text{r}}-2m)m} \frac{{a}_{m,n}}{n{!}} \Upsilon \left( n+1, \frac{mx}{{\bar {\gamma }}_{\text{r}}} \right) .\end{eqnarray*}

Similarly, the eavesdropper can estimate the symbol *s* as

\begin{eqnarray*}{\mathbf{z}}_{\text{e}}^{\dagger }{\mathbf{y}}_{\text{e}}={\mathbf{z}}_{\text{e}}^{\dagger }{\mathbf{H}}_{\text{e}}{\mathbf{w}}_{\text{t}}s+{\mathbf{z}}_{\text{e}}^{\dagger }{\mathbf{n}}_{\text{e}} \end{eqnarray*}

where the receive weight vector

\begin{eqnarray*}{\mathbf{z}}_{\text{e}}= \frac{{\mathbf{H}}_{\text{e}}{\mathbf{w}}_{\text{t}}}{\parallel {\mathbf{H}}_{\text{e}}{\mathbf{w}}_{\text{t}}\parallel } \end{eqnarray*}

is chosen to maximize the SNR of the estimate, yielding

(8) \begin{eqnarray*}{\gamma }_{\text{e,TR}}={\bar {\gamma }}_{\text{e}}\parallel {\mathbf{H}}_{\text{e}}{\mathbf{w}}_{\text{t}}{\parallel }^{2}\end{eqnarray*}

where ${\bar {\gamma }}_{\text{e}}= \frac{P}{{\sigma }_{\text{e}}^{2}} $ is the average SNR at the eavesdropper. The probability density function (PDF) of *γ*_e,TR_ in Eq. (8) is given by Maichalernnukul (2018)

(9) \begin{eqnarray*}{f}_{{\gamma }_{\text{e,TR}}}(x)= \frac{{x}^{{M}_{\text{e}}-1}{e}^{- \frac{x}{{\bar {\gamma }}_{\text{e}}} }}{({M}_{\text{e}}-1){!}{{\bar {\gamma }}_{\text{e}}}^{{M}_{\text{e}}}} .\end{eqnarray*}

### Spatial multiplexing system

Unlike the transmit-receive diversity system, the spatial multiplexing system allows the simultaneous transmission of different symbols, i.e., the *i*th antenna (*i* = 1, 2, …, *M*_t_) at the transmitter is used to transmit the symbol *s*_*i*_ ∈ ℂ (with E[|*s*_*i*_|^2^] = *P*) . Let **s** = [*s*_1_, *s*_2_, …, *s*_*M*_t__]^T^. The received signal vectors at the legitimate receiver and the passive eavesdropper are given, respectively, by

\begin{eqnarray*}{\mathbf{y}}_{\text{r}}={\mathbf{H}}_{\text{r}}\mathbf{s}+{\mathbf{n}}_{\text{r}} \end{eqnarray*}

where **H**_r_ and **n**_r_ are defined in Eq. (2), and

\begin{eqnarray*}{\mathbf{y}}_{\text{e}}={\mathbf{H}}_{\text{e}}\mathbf{s}+{\mathbf{n}}_{\text{e}} \end{eqnarray*}

where **H**_e_ and **n**_e_ are defined in Eq. (3). We assume that the receiver and the eavesdropper know **H**_r_ and **H**_e_, respectively, and the numbers of antennas at these two terminals (*M*_r_ and *M*_e_) are no less than the number of antennas at the transmitter (*M*_t_). The assumption on *M*_t_, *M*_r_, and *M*_e_ is necessary for the theoretical analysis hereafter.

In order for the receiver to estimate **s**, the ZF or MMSE receive weight (equalizing) matrix is applied to **y**_r_. These matrices are given by Tse & Viswanath (2005)

\begin{eqnarray*}{\mathbf{W}}_{\text{r,ZF}}={ \left( {\mathbf{H}}_{\text{r}}^{\dagger }{\mathbf{H}}_{\text{r}} \right) }^{-1}{\mathbf{H}}_{\text{r}}^{\dagger } \end{eqnarray*}

and

\begin{eqnarray*}{\mathbf{W}}_{\text{r,MMSE}}={ \left( {\mathbf{H}}_{\text{r}}^{\dagger }{\mathbf{H}}_{\text{r}}+ \frac{1}{{\bar {\gamma }}_{\text{r}}} {\mathbf{I}}_{{M}_{\text{t}}} \right) }^{-1}{\mathbf{H}}_{\text{r}}^{\dagger }. \end{eqnarray*}

It is noteworthy that as the average SNR at the receiver grows very large, i.e., ${\bar {\gamma }}_{\text{r}}\rightarrow \infty $, **W**_r,MMSE_ approaches **W**_r,ZF_. Left multiplying **y**_r_ by **W**_r,ZF_ and **W**_r,MMSE_, we obtain the *i*th symbol estimate (*i* = 1, 2, …, *M*_t_), the SNRs of which are, respectively, (Jiang, Varanasi & Li, 2011)

(10) \begin{eqnarray*}{\gamma }_{\text{r,ZF},i}= \frac{{\bar {\gamma }}_{\text{r}}}{{ \left[ { \left( {\mathbf{H}}_{\text{r}}^{\dagger }{\mathbf{H}}_{\text{r}} \right) }^{-1} \right] }_{ii}} \end{eqnarray*}

and

(11) \begin{eqnarray*}{\gamma }_{\text{r,MMSE},i}= \frac{{\bar {\gamma }}_{\text{r}}}{{ \left[ { \left( {\mathbf{H}}_{\text{r}}^{\dagger }{\mathbf{H}}_{\text{r}}+ \frac{1}{{\bar {\gamma }}_{\text{r}}} {\mathbf{I}}_{{M}_{\text{t}}} \right) }^{-1} \right] }_{ii}} -1.\end{eqnarray*}

The CDFs of *γ*_r,ZF,*i*_ and *γ*_r,MMSE,*i*_ are given, respectively, by Chen & Wang (2007)

(12) \begin{eqnarray*}{F}_{{\gamma }_{\text{r,ZF}}}(x)=1-{e}^{- \frac{x}{{\bar {\gamma }}_{\text{r}}} }\sum _{m=0}^{{M}_{\text{r}}-{M}_{\text{t}}} \frac{{x}^{m}}{m{!}{\bar {\gamma }}_{\text{r}}^{m}} \end{eqnarray*}

and Smith (2007)

(13) \begin{eqnarray*}{F}_{{\gamma }_{\text{r,MMSE}}}(x)=1- \frac{{e}^{- \frac{x}{{\bar {\gamma }}_{\text{r}}} }}{(x+1)^{{M}_{\text{t}}-1}} \sum _{m=0}^{{M}_{\text{r}}-1}{d}_{m}{x}^{m}\end{eqnarray*}

where ${d}_{m}={\mathop{\sum }\nolimits }_{n=\max (0,m-{M}_{\text{t}}+1)}^{m} \left( {{M}_{\text{t}}-1\atop m-n} \right) \frac{1}{n{!}{\bar {\gamma }}_{\text{r}}^{n}} $. The symbol index *i* is omitted from Eqs. (12) and (13) because all the elements of **H**_r_ are statistically independent and identically distributed.

Similarly, the eavesdropper performs ZF or MMSE equalization, and the resulting SNRs of the *i*th symbol estimate (i.e., *γ*_e,ZF,*i*_ and *γ*_e,MMSE,*i*_) can be expressed, respectively, as Eqs. (10) and (11) with the subscript r being replaced by the subscript e. Replacing the subscript r with the subscript e in Eqs. (12) and (13), and taking the derivative of these equations with respect to *x*, we obtain the PDFs for *γ*_e,ZF,*i*_ and *γ*_e,MMSE,*i*_, respectively, as

(14) \begin{eqnarray*}{f}_{{\gamma }_{\text{e,ZF}}}(x)= \frac{{x}^{{M}_{\text{e}}-{M}_{\text{t}}}{e}^{- \frac{x}{{\bar {\gamma }}_{\text{e}}} }}{({M}_{\text{e}}-{M}_{\text{t}}){!}{\bar {\gamma }}_{\text{e}}^{{M}_{\text{e}}-{M}_{\text{t}}+1}} \end{eqnarray*}

and

(15) \begin{eqnarray*}{f}_{{\gamma }_{\text{e,MMSE}}}(x)= \frac{{e}^{- \frac{x}{{\bar {\gamma }}_{\text{e}}} }}{(x+1)^{{M}_{\text{t}}}} \sum _{m=0}^{{M}_{\text{e}}-1}{g}_{m} \left[ \frac{{x}^{m+1}}{{\bar {\gamma }}_{\text{e}}} + \left( {M}_{\text{t}}+ \frac{1}{{\bar {\gamma }}_{\text{e}}} -m-1 \right) {x}^{m}-m{x}^{m-1} \right] \end{eqnarray*}

where *g*_*m*_ is similar to *d*_*m*_, except that the subscript r is replaced by the subscript e.

## Exact Secrecy Outage Probability

The secrecy outage probability is defined as the probability that the instantaneous secrecy capacity is less than a target secrecy rate *R* > 0 (Bloch et al., 2008). From Eq. (1), this performance metric can be expressed as

(16) \begin{eqnarray*}{P}_{\text{out}}(R)& =& \text{Pr} \left\{ {C}_{\text{s}}\lt R \right\} \nonumber\\\displaystyle & =& \text{Pr} \left\{ {\gamma }_{\text{r}}\lt {2}^{R}{\gamma }_{\text{e}}+{2}^{R}-1 \right\} \nonumber\\\displaystyle & =& \int \nolimits \nolimits _{0}^{\infty }{f}_{{\gamma }_{\text{e}}}(v)\,{F}_{{\gamma }_{\text{r}}} \left( {2}^{R}v+{2}^{R}-1 \right) \text{d}v.\end{eqnarray*}

### Transmit-receive diversity system

From Eqs. (7), (9) and (16), we can derive the exact secrecy outage probability for the transmit-receive diversity system as follows:

(17) \begin{eqnarray*}{P}_{\text{out,TR}}(R)& =& \frac{1}{({M}_{\text{e}}-1){!}{{\bar {\gamma }}_{\text{e}}}^{{M}_{\text{e}}}} \sum _{m=1}^{L}\sum _{n= \left\vert {M}_{\text{t}}-{M}_{\text{r}} \right\vert }^{({M}_{\text{t}}+{M}_{\text{r}}-2m)m} \frac{{a}_{m,n}}{n{!}} \int \nolimits \nolimits _{0}^{\infty }{v}^{{M}_{\text{e}}-1}{e}^{- \frac{v}{{\bar {\gamma }}_{\text{e}}} }\nonumber\\\displaystyle & & \times \Upsilon \left( n+1, \frac{({2}^{R}v+{2}^{R}-1)m}{{\bar {\gamma }}_{\text{r}}} \right) \text{d}v\nonumber\\\displaystyle & =& \frac{1}{({M}_{\text{e}}-1){!}{{\bar {\gamma }}_{\text{e}}}^{{M}_{\text{e}}}} \sum _{m=1}^{L}\sum _{n= \left\vert {M}_{\text{t}}-{M}_{\text{r}} \right\vert }^{({M}_{\text{t}}+{M}_{\text{r}}-2m)m}{a}_{m,n} \left[ \right. \int \nolimits \nolimits _{0}^{\infty }{v}^{{M}_{\text{e}}-1}{e}^{- \frac{v}{{\bar {\gamma }}_{\text{e}}} }\text{d}v\nonumber\\\displaystyle & & -{e}^{- \frac{({2}^{R}-1)m}{{\bar {\gamma }}_{\text{r}}} }\sum _{k=0}^{n}{ \left( \frac{m}{{\bar {\gamma }}_{\text{r}}} \right) }^{k}\sum _{l=0}^{k} \frac{{2}^{lR}({2}^{R}-1)^{k-l}}{l{!}(k-l){!}} \int \nolimits \nolimits _{0}^{\infty }{v}^{l+{M}_{\text{e}}-1}{e}^{- \left( \frac{{2}^{R}m}{{\bar {\gamma }}_{\text{r}}} + \frac{1}{{\bar {\gamma }}_{\text{e}}} \right) v}\text{d}v \left( \right. \nonumber\\\displaystyle & =& 1- \frac{1}{({M}_{\text{e}}-1){!}{{\bar {\gamma }}_{\text{e}}}^{{M}_{\text{e}}}} \sum _{m=1}^{L}\sum _{n= \left\vert {M}_{\text{t}}-{M}_{\text{r}} \right\vert }^{({M}_{\text{t}}+{M}_{\text{r}}-2m)m}{a}_{m,n}{e}^{- \frac{({2}^{R}-1)m}{{\bar {\gamma }}_{\text{r}}} }\sum _{k=0}^{n}{ \left( \frac{m}{{\bar {\gamma }}_{\text{r}}} \right) }^{k}\nonumber\\\displaystyle & & \times \sum _{l=0}^{k} \frac{ \left( l+{M}_{\text{e}}-1 \right) {!}\,{2}^{lR}({2}^{R}-1)^{k-l}}{l{!}(k-l){!}{ \left( \frac{{2}^{R}m}{{\bar {\gamma }}_{\text{r}}} + \frac{1}{{\bar {\gamma }}_{\text{e}}} \right) }^{l+{M}_{\text{e}}}} \end{eqnarray*}

where the second equality is obtained by using (Gradshteyn & Ryzhik, 2000, Equations (1.111) and (8.352.1)), and the last equality is obtained by using (Gradshteyn & Ryzhik, 2000, Equation (3.351.3)) and (Maaref & Aïssa, 2005, Equation (11)). For the special case of *M*_t_ = *M*_r_ = *M*_e_ = 1, the secrecy outage probability expression in Eq. (17) reduces to

(18) \begin{eqnarray*}{P}_{\text{out,TR}}(R)=1- \frac{{\bar {\gamma }}_{\text{r}}\,{e}^{- \frac{{2}^{R}-1}{{\bar {\gamma }}_{\text{r}}} }}{{\bar {\gamma }}_{\text{r}}+{2}^{R}{\bar {\gamma }}_{\text{e}}} \end{eqnarray*}

which agrees exactly with a result given in (Bloch et al., 2008, Equation (9)).

### Spatial multiplexing system

From Eqs. (12), (14) and (16), we can derive the exact secrecy outage probability for the spatial multiplexing system with ZF equalization as follows:

(19) \begin{eqnarray*}{P}_{\text{out,ZF}}(R)& =& \int \nolimits \nolimits _{0}^{\infty }{f}_{{\gamma }_{\text{e,ZF}}}(v)\text{d}v- \frac{{e}^{- \frac{{2}^{R}-1}{{\bar {\gamma }}_{\text{r}}} }}{({M}_{\text{e}}-{M}_{\text{t}}){!}{{\bar {\gamma }}_{\text{e}}}^{{M}_{\text{e}}-{M}_{\text{t}}+1}} \sum _{m=0}^{{M}_{\text{r}}-{M}_{\text{t}}} \frac{1}{m{!}{\bar {\gamma }}_{\text{r}}^{m}} \nonumber\\\displaystyle & & \times \int \nolimits \nolimits _{0}^{\infty }({2}^{R}v+{2}^{R}-1)^{m}{v}^{{M}_{\text{e}}-{M}_{\text{t}}}{e}^{- \left( \frac{{2}^{R}}{{\bar {\gamma }}_{\text{r}}} + \frac{1}{{\bar {\gamma }}_{\text{e}}} \right) v}\text{d}v\nonumber\\\displaystyle & =& 1- \frac{{e}^{- \frac{{2}^{R}-1}{{\bar {\gamma }}_{\text{r}}} }}{({M}_{\text{e}}-{M}_{\text{t}}){!}{{\bar {\gamma }}_{\text{e}}}^{{M}_{\text{e}}-{M}_{\text{t}}+1}} \sum _{m=0}^{{M}_{\text{r}}-{M}_{\text{t}}} \frac{1}{{\bar {\gamma }}_{\text{r}}^{m}} \sum _{n=0}^{m} \frac{{2}^{nR}({2}^{R}-1)^{m-n}}{n{!}(m-n){!}} \nonumber\\\displaystyle & & \times \int \nolimits \nolimits _{0}^{\infty }{v}^{n+{M}_{\text{e}}-{M}_{\text{t}}}{e}^{- \left( \frac{{2}^{R}}{{\bar {\gamma }}_{\text{r}}} + \frac{1}{{\bar {\gamma }}_{\text{e}}} \right) v}\text{d}v\nonumber\\\displaystyle & =& 1- \frac{{e}^{- \frac{{2}^{R}-1}{{\bar {\gamma }}_{\text{r}}} }}{({M}_{\text{e}}-{M}_{\text{t}}){!}{ \left( \frac{{2}^{R}{\bar {\gamma }}_{\text{e}}}{{\bar {\gamma }}_{\text{r}}} +1 \right) }^{{M}_{\text{e}}-{M}_{\text{t}}+1}} \nonumber\\\displaystyle & & \times \sum _{m=0}^{{M}_{\text{r}}-{M}_{\text{t}}} \frac{1}{{\bar {\gamma }}_{\text{r}}^{m}} \sum _{n=0}^{m} \frac{{2}^{nR}({2}^{R}-1)^{m-n}(n+{M}_{\text{e}}-{M}_{\text{t}}){!}}{n{!}(m-n){!}{ \left( \frac{{2}^{R}}{{\bar {\gamma }}_{\text{r}}} + \frac{1}{{\bar {\gamma }}_{\text{e}}} \right) }^{n}} \end{eqnarray*}

where the second equality is obtained by using (Gradshteyn & Ryzhik, 2000, Equation (1.111)) and (Papoulis & Pillai, 2002, Equation (4-18)), and the last equality is obtained by using (Gradshteyn & Ryzhik, 2000, Equation (3.351.3)). For the special case of *M*_t_ = *M*_r_ = *M*_e_ = 1, Eq. (19) simplifies to Eq. (18).

Meanwhile, the secrecy outage probability for the spatial multiplexing system with MMSE equalization can be derived from Eqs. (13), (15) and (16) as follows:

(20) \begin{eqnarray*}{P}_{\text{out,MMSE}}(R)& =& \int \nolimits \nolimits _{0}^{\infty }{f}_{{\gamma }_{\text{e,MMSE}}}(v)\,\text{d}v- \frac{{e}^{- \frac{{2}^{R}-1}{{\bar {\gamma }}_{\text{r}}} }}{{2}^{({M}_{\text{t}}-1)R}} \sum _{m=0}^{{M}_{\text{e}}-1}{g}_{m}\sum _{n=0}^{{M}_{\text{r}}-1}{d}_{n}\nonumber\\\displaystyle & & \times \, \left[ \right. \int \nolimits \nolimits _{0}^{\infty } \frac{({2}^{R}v+{2}^{R}-1)^{n}{e}^{- \left( \frac{{2}^{R}}{{\bar {\gamma }}_{\text{r}}} + \frac{1}{{\bar {\gamma }}_{\text{e}}} \right) v}}{(v+1)^{2{M}_{\text{t}}-1}} \nonumber\\\displaystyle & & \times \left[ \right. \frac{{v}^{m+1}}{{\bar {\gamma }}_{\text{e}}} + \left( {M}_{\text{t}}+ \frac{1}{{\bar {\gamma }}_{\text{e}}} -m-1 \right) {v}^{m}-m{v}^{m-1} \left( \right. \text{d}v \left( \right. \nonumber\\\displaystyle & =& 1- \frac{{e}^{ \frac{1}{{\bar {\gamma }}_{\text{r}}} + \frac{1}{{\bar {\gamma }}_{\text{e}}} }}{{2}^{({M}_{\text{t}}-1)R}} \sum _{m=0}^{{M}_{\text{e}}-1}{g}_{m}\sum _{n=0}^{{M}_{\text{r}}-1}{d}_{n}\sum _{k=0}^{n} \left( {n\atop k} \right) (-1)^{k}{2}^{(n-k)R}\nonumber\\\displaystyle & & \times \left[ \right. \frac{1}{{\bar {\gamma }}_{\text{e}}} \sum _{{l}_{1}=0}^{m+1} \left( {m+1\atop {l}_{1}} \right) (-1)^{{l}_{1}}\int \nolimits \nolimits _{1}^{\infty }{v}^{m+n-k-{l}_{1}-2{M}_{\text{t}}+2}{e}^{- \left( \frac{{2}^{R}}{{\bar {\gamma }}_{\text{r}}} + \frac{1}{{\bar {\gamma }}_{\text{e}}} \right) v}\text{d}v\nonumber\\\displaystyle & & + \left( {M}_{\text{t}}+ \frac{1}{{\bar {\gamma }}_{\text{e}}} -m-1 \right) \sum _{{l}_{2}=0}^{m} \left( {m\atop {l}_{2}} \right) (-1)^{{l}_{2}}\int \nolimits \nolimits _{1}^{\infty }{v}^{m+n-k-{l}_{2}-2{M}_{\text{t}}+1}{e}^{- \left( \frac{{2}^{R}}{{\bar {\gamma }}_{\text{r}}} + \frac{1}{{\bar {\gamma }}_{\text{e}}} \right) v}\text{d}v\nonumber\\\displaystyle & & +m\sum _{{l}_{3}=0}^{m-1} \left( {m-1\atop {l}_{3}} \right) (-1)^{{l}_{3}}\int \nolimits \nolimits _{1}^{\infty }{v}^{m+n-k-{l}_{3}-2{M}_{\text{t}}}{e}^{- \left( \frac{{2}^{R}}{{\bar {\gamma }}_{\text{r}}} + \frac{1}{{\bar {\gamma }}_{\text{e}}} \right) v}\,\text{d}v \left( \right. \nonumber\\\displaystyle & =& 1- \frac{{e}^{ \frac{1}{{\bar {\gamma }}_{\text{r}}} + \frac{1}{{\bar {\gamma }}_{\text{e}}} }}{{2}^{({M}_{\text{t}}-1)R}} \sum _{m=0}^{{M}_{\text{e}}-1}{g}_{m}\sum _{n=0}^{{M}_{\text{r}}-1}{d}_{n}\sum _{k=0}^{n} \left( {n\atop k} \right) (-1)^{k}{2}^{(n-k)R}\nonumber\\\displaystyle & & \times \left[ \right. \frac{1}{{\bar {\gamma }}_{\text{e}}} \sum _{{l}_{1}=0}^{m+1} \left( {m+1\atop {l}_{1}} \right) \frac{(-1)^{{l}_{1}}\Gamma \left( m+n-k-{l}_{1}-2{M}_{\text{t}}+3, \frac{{2}^{R}}{{\bar {\gamma }}_{\text{r}}} + \frac{1}{{\bar {\gamma }}_{\text{e}}} \right) }{{ \left( \frac{{2}^{R}}{{\bar {\gamma }}_{\text{r}}} + \frac{1}{{\bar {\gamma }}_{\text{e}}} \right) }^{m+n-k-{l}_{1}-2{M}_{\text{t}}+3}} \nonumber\\\displaystyle & & + \left( {M}_{\text{t}}+ \frac{1}{{\bar {\gamma }}_{\text{e}}} -m-1 \right) \sum _{{l}_{2}=0}^{m} \left( {m\atop {l}_{2}} \right) \frac{(-1)^{{l}_{2}}\Gamma \left( m+n-k-{l}_{2}-2{M}_{\text{t}}+2, \frac{{2}^{R}}{{\bar {\gamma }}_{\text{r}}} + \frac{1}{{\bar {\gamma }}_{\text{e}}} \right) }{{ \left( \frac{{2}^{R}}{{\bar {\gamma }}_{\text{r}}} + \frac{1}{{\bar {\gamma }}_{\text{e}}} \right) }^{m+n-k-{l}_{2}-2{M}_{\text{t}}+2}} \nonumber\\\displaystyle & & +m\sum _{{l}_{3}=0}^{m-1} \left( {m-1\atop {l}_{3}} \right) \frac{(-1)^{{l}_{3}}\Gamma \left( m+n-k-{l}_{3}-2{M}_{\text{t}}+1, \frac{{2}^{R}}{{\bar {\gamma }}_{\text{r}}} + \frac{1}{{\bar {\gamma }}_{\text{e}}} \right) }{{ \left( \frac{{2}^{R}}{{\bar {\gamma }}_{\text{r}}} + \frac{1}{{\bar {\gamma }}_{\text{e}}} \right) }^{m+n-k-{l}_{3}-2{M}_{\text{t}}+1}} \left( \right. \end{eqnarray*}

where the second equality is obtained by changing the limits of integration and using (Gradshteyn & Ryzhik, 2000, Equation (1.111)) and (Papoulis & Pillai, 2002, Equation (4-18)), and the last equality is obtained by using (Gradshteyn & Ryzhik, 2000, Equation (3.381.3)). For the special case of *M*_t_ = *M*_r_ = *M*_e_ = 1, Eq. (20) reduces to Eq. (18).

## Asymptotic Secrecy Outage Probability

In this section, we focus on deriving the asymptotic secrecy outage probability of the aforementioned systems as ${\bar {\gamma }}_{\text{r}}\rightarrow \infty $. This expression enables one to analyze the secrecy performance in the high-SNR regime through two performance indicators: secrecy diversity order and secrecy array gain (Yang et al., 2013). The secrecy diversity order indicates the slope of the secrecy outage probability versus ${\bar {\gamma }}_{\text{r}}$ curve at high SNR in a log–log scale, whereas the secrecy array gain indicates the shift of the curve with respect to the benchmark secrecy outage curve.

### Transmit-receive diversity system

First, we look for a first-order expansion of Eq. (5), which will be immediate from a first-order expansion of det(**S**(*x*)). Following the approach outlined in (McKay, 2006, Appendix B.7) and using (Kalman, 1984, Equations (1) and (2)), it is straightforward to show that the first-order Taylor expansion of det(**S**(*x*)) around *x* = 0 is

(21) \begin{eqnarray*}\det \nolimits (\mathbf{S}(x))= \left[ \right. \prod _{p=1}^{L} \frac{(K-p){!}\,[(L-p){!}]^{2}}{({M}_{\text{t}}+{M}_{\text{r}}-p){!}} \left( \right. {x}^{{M}_{\text{t}}{M}_{\text{r}}}+o \left( {x}^{{M}_{\text{t}}{M}_{\text{r}}} \right) .\end{eqnarray*}

Substituting Eq. (21) into Eq. (5) yields

(22) \begin{eqnarray*}{F}_{\lambda }(x)= \left[ \right. \prod _{p=1}^{L} \frac{(L-p){!}}{({M}_{\text{t}}+{M}_{\text{r}}-p){!}} \left( \right. {x}^{{M}_{\text{t}}{M}_{\text{r}}}+o \left( {x}^{{M}_{\text{t}}{M}_{\text{r}}} \right) .\end{eqnarray*}

Using Eq. (22) and (Papoulis & Pillai, 2002, Example 5-1), the first-order expansion of the CDF of *γ*_r,TR_ is given by

(23) \begin{eqnarray*}{F}_{{\gamma }_{\text{r,TR}}}(x)= \left[ \right. \prod _{p=1}^{L} \frac{(L-p){!}}{({M}_{\text{t}}+{M}_{\text{r}}-p){!}} \left( \right. { \left( \frac{x}{{\bar {\gamma }}_{\text{r}}} \right) }^{{M}_{\text{t}}{M}_{\text{r}}}+o \left( { \left( \frac{x}{{\bar {\gamma }}_{\text{r}}} \right) }^{{M}_{\text{t}}{M}_{\text{r}}} \right) .\end{eqnarray*}

Using Eqs. (9), (16) and (23), and following the same procedure as used in Eq. (17), an asymptotic expression for *P*_out,TR_(*R*) with ${\bar {\gamma }}_{\text{r}}\rightarrow \infty $ is obtained as

(24) \begin{eqnarray*}{P}_{\text{out,TR}}^{\infty }(R)={ \left( {A}_{\text{TR}}{\bar {\gamma }}_{\text{r}} \right) }^{-{D}_{\text{TR}}}+o \left( {\bar {\gamma }}_{\text{r}}^{-{D}_{\text{TR}}} \right) \end{eqnarray*}

where the secrecy diversity gain is

(25) \begin{eqnarray*}{D}_{\text{TR}}={M}_{\text{t}}{M}_{\text{r}}\end{eqnarray*}

and the secrecy array gain is

(26) \begin{eqnarray*}{A}_{\text{TR}}& =& \left[ \right. \frac{1}{({M}_{\text{e}}-1){!}} \left[ \right. \prod _{p=1}^{L} \frac{(L-p){!}}{({M}_{\text{t}}+{M}_{\text{r}}-p){!}} \left( \right. \sum _{n=0}^{{M}_{\text{t}}{M}_{\text{r}}} \left( {{M}_{\text{t}}{M}_{\text{r}}\atop n} \right) \nonumber\\\displaystyle & & \times (n+{M}_{\text{e}}-1){!}{2}^{nR}({2}^{R}-1)^{{M}_{\text{t}}{M}_{\text{r}}-n}{\bar {\gamma }}_{\text{e}}^{n}{ \left( \right. }^{- \frac{1}{{M}_{\text{t}}{M}_{\text{r}}} }.\end{eqnarray*}

It is clear from Eq. (25) that the secrecy diversity order is dependent on *M*_t_ and *M*_r_, and independent of *M*_e_. It can also be seen from Eq. (26) that the eavesdropper channel has an adverse impact on the secrecy array gain. Accordingly, increasing the number of antennas at the eavesdropper lessens the secrecy array gain, thereby rising the secrecy outage probability.

### Spatial multiplexing system

Applying (Gradshteyn & Ryzhik, 2000, Equation (1.211.1)) to the exponential function in Eq. (12) and performing some algebraic manipulations, the first-order expansion of the CDF of *γ*_r,ZF,*i*_ can be derived as

(27) \begin{eqnarray*}{F}_{{\gamma }_{\text{r,ZF}}}(x)= \frac{{x}^{{M}_{\text{r}}-{M}_{\text{t}}+1}}{({M}_{\text{r}}-{M}_{\text{t}}+1){!}{\bar {\gamma }}_{\text{r}}^{{M}_{\text{r}}-{M}_{\text{t}}+1}} +o \left( { \left( \frac{x}{{\bar {\gamma }}_{\text{r}}} \right) }^{{M}_{\text{r}}-{M}_{\text{t}}+1} \right) .\end{eqnarray*}

Using Eqs. (14), (16) and (27), and following the same procedure as used in Eq. (19), an asymptotic expression for *P*_out,ZF_(*R*) with ${\bar {\gamma }}_{\text{r}}\rightarrow \infty $ is obtained as

(28) \begin{eqnarray*}{P}_{\text{out,ZF}}^{\infty }(R)=({A}_{\text{ZF}}{\bar {\gamma }}_{\text{r}})^{-{D}_{\text{ZF}}}+o \left( {\bar {\gamma }}_{\text{r}}^{-{D}_{\text{ZF}}} \right) \end{eqnarray*}

where

(29) \begin{eqnarray*}{D}_{\text{ZF}}={M}_{\text{r}}-{M}_{\text{t}}+1\end{eqnarray*}

and

(30) \begin{eqnarray*}{A}_{\text{ZF}}={ \left[ \frac{\sum _{n=0}^{{M}_{\text{r}}-{M}_{\text{t}}+1} \left( {{M}_{\text{r}}-{M}_{\text{t}}+1\atop n} \right) {2}^{nR}({2}^{R}-1)^{{M}_{\text{r}}-{M}_{\text{t}}+1-n}(n+{M}_{\text{e}}-{M}_{\text{t}}){!}{\bar {\gamma }}_{\text{e}}^{n}}{({M}_{\text{r}}-{M}_{\text{t}}+1){!}\,({M}_{\text{e}}-{M}_{\text{t}}){!}} \right] }^{- \frac{1}{{M}_{\text{r}}-{M}_{\text{t}}+1} }.\end{eqnarray*}

Adopting the same steps as for deriving the first-order expansion of *F*_*γ*_r,ZF__(*x*), we obtain

(31) \begin{eqnarray*}{F}_{{\gamma }_{\text{r,MMSE}}}(x)= \frac{{x}^{{M}_{\text{r}}}}{({M}_{\text{r}}-{M}_{\text{t}}+1){!}{\bar {\gamma }}_{\text{r}}^{{M}_{\text{r}}-{M}_{\text{t}}+1}(x+1)^{{M}_{\text{t}}-1}} +\,o \left( { \left( \frac{x}{{\bar {\gamma }}_{\text{r}}} \right) }^{{M}_{\text{r}}-{M}_{\text{t}}+1} \right) .\end{eqnarray*}

Using Eqs. (15), (16) and (31), and following the same procedure as used in Eq. (20), an asymptotic expression for *P*_out,MMSE_(*R*) with ${\bar {\gamma }}_{\text{r}}\rightarrow \infty $ is obtained as

(32) \begin{eqnarray*}{P}_{\text{out,MMSE}}^{\infty }(R)=({A}_{\text{MMSE}}{\bar {\gamma }}_{\text{r}})^{-{D}_{\text{MMSE}}}+o \left( {\bar {\gamma }}_{\text{r}}^{-{D}_{\text{MMSE}}} \right) \end{eqnarray*}

where

(33) \begin{eqnarray*}{D}_{\text{MMSE}}={M}_{\text{r}}-{M}_{\text{t}}+1\end{eqnarray*}

and

(34) \begin{eqnarray*}{A}_{\text{MMSE}}& =& \left[ \right. \frac{{e}^{ \frac{1}{{\bar {\gamma }}_{\text{e}}} }{2}^{({M}_{\text{r}}-{M}_{\text{t}}+1)R}}{({M}_{\text{r}}-{M}_{\text{t}}+1){!}} \sum _{m=0}^{{M}_{\text{e}}-1}{g}_{m}\sum _{n=0}^{{M}_{\text{r}}} \left( {{M}_{\text{r}}\atop n} \right) \frac{(-1)^{n}{\bar {\gamma }}_{\text{e}}^{m-n+{M}_{\text{r}}-2{M}_{\text{t}}+1}}{{2}^{nR}} \nonumber\\\displaystyle & & \times \left[ \right. {\bar {\gamma }}_{\text{e}}\sum _{{k}_{1}=0}^{m+1} \left( {m+1\atop {k}_{1}} \right) { \left( - \frac{1}{{\bar {\gamma }}_{\text{e}}} \right) }^{{k}_{1}}\Gamma \left( m-n-{k}_{1}+{M}_{\text{r}}-2{M}_{\text{t}}+3, \frac{1}{{\bar {\gamma }}_{\text{e}}} \right) \nonumber\\\displaystyle & & +{\bar {\gamma }}_{\text{e}} \left( {M}_{\text{t}}+ \frac{1}{{\bar {\gamma }}_{\text{e}}} -m-1 \right) \sum _{{k}_{2}=0}^{m} \left( {m\atop {k}_{2}} \right) { \left( - \frac{1}{{\bar {\gamma }}_{\text{e}}} \right) }^{{k}_{2}}\Gamma \left( m-n-{k}_{2}+{M}_{\text{r}}-2{M}_{\text{t}}+2, \frac{1}{{\bar {\gamma }}_{\text{e}}} \right) \nonumber\\\displaystyle & & -m\sum _{{k}_{3}=0}^{m-1} \left( {m-1\atop {k}_{3}} \right) { \left( - \frac{1}{{\bar {\gamma }}_{\text{e}}} \right) }^{{k}_{3}}\Gamma \left( m-n-{k}_{3}+{M}_{\text{r}}-2{M}_{\text{t}}+1, \frac{1}{{\bar {\gamma }}_{\text{e}}} \right) \left( \right. { \left( \right. }^{- \frac{1}{{M}_{\text{r}}-{M}_{\text{t}}+1} }.\end{eqnarray*}

It is obvious from Eqs. (29) and (33) that the secrecy diversity orders of the spatial multiplexing systems with ZF equalization and MMSE equalization are dependent on *M*_t_ and *M*_r_, and independent of *M*_e_. It can also be observed from Eqs. (30) and (34) that increasing *M*_e_ decreases the corresponding secrecy array gains.

## Numerical Results

**Figure 1:**
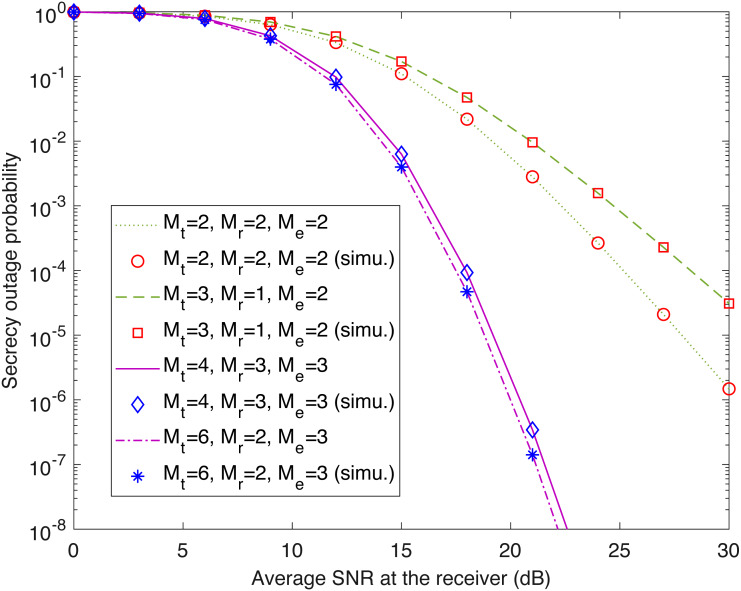
Secrecy outage probability of transmit-receive diversity system (*P*_out,TR_) as a function of ${\bar {\gamma }}_{\text{r}}$. This figure shows the theoretical and simulated secrecy outage curves for the transmit-receive diversity system with different numbers of antennas at the transmitter (*M*_t_), the legitimate receiver (*M*_r_), and the eavesdropper (*M*_e_). The simulation results are labeled with “simu.”.

In this section, we validate the preceding theoretical analysis and investigate the effect of the various system parameters. For these purposes, theoretical and simulation results are obtained by using MATLAB. Specifically, we use the closed-form expressions derived above to generate the theoretical results, and adopt the Monte Carlo method to generate the simulation results. Remember that ${\bar {\gamma }}_{\text{r}}$ and ${\bar {\gamma }}_{\text{e}}$ are the average SNRs at the legitimate receiver and the passive eavesdropper, respectively. Unless otherwise indicated, the SNR ${\bar {\gamma }}_{\text{e}}$ is set to 10 dB, and the target secrecy rate *R* is set to 1 bit/s/Hz. Figure 1 shows the theoretical secrecy outage probability of the transmit-receive diversity system (computed with Eq. (17)) and its simulation counterpart (labeled with “simu.”) against ${\bar {\gamma }}_{\text{r}}$. As seen in the figure, the theoretical and simulation results match perfectly. For a given ${\bar {\gamma }}_{\text{r}}$, when *M*_t_ + *M*_r_ = 4 and *M*_e_ = 2, the secrecy outage probability with *M*_t_ = 2 and *M*_r_ = 2 is lower than that with *M*_t_ = 3 and *M*_r_ = 1. This is consistent with the fact that for a fixed total number of antennas at the transmitter and legitimate receiver (*M*_t_ + *M*_r_), a more-balanced antenna configuration provides a larger diversity gain (Dighe, Mallik & Jamuar, 2003; Maaref & Aïssa, 2005). Specifically, from Eq. (25), we have *D*_TR_ = 4 for *M*_t_ = 2 and *M*_r_ = 2, and *D*_TR_ = 3 for *M*_t_ = 3 and *M*_r_ = 1. However, when *M*_t_*M*_r_ = 12 and *M*_e_ = 3, the secrecy outage probability with *M*_t_ = 4 and *M*_r_ = 3 is higher than that with *M*_t_ = 6 and *M*_r_ = 2. The reason is that for the same product of *M*_t_ and *M*_r_, an increase in *M*_t_ + *M*_r_ yields a performance enhancement (Dighe, Mallik & Jamuar, 2003).

**Figure 2:**
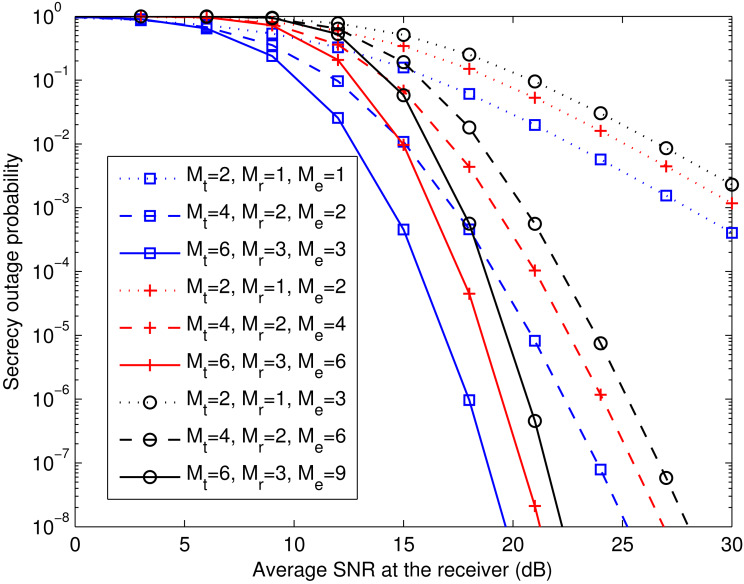
*P*_out,TR_ for different combinations of *M*_t_, *M*_r_, and *M*_e_. This figure shows the theoretical secrecy outage curves for the transmit-receive diversity system, comparing different numbers of antennas at the transmitter (*M*_t_), the legitimate receiver (*M*_r_), and the eavesdropper (*M*_e_).

Figure 2 depicts the theoretical secrecy outage probability of the aforementioned system for different combinations of *M*_t_, *M*_r_, and *M*_e_. We observe that when (*M*_t_, *M*_r_) is kept fixed (i.e., at (2, 1), (4, 2), or (6, 3)), the larger *M*_e_ is, the smaller the array gain (as discussed in Eq. (26)), which worsens the secrecy outage performance. Furthermore, it can be seen that for a given ${\bar {\gamma }}_{\text{r}}$, the secrecy outage probability with (*M*_t_, *M*_r_, *M*_e_) = (2, 1, 1) is higher than that with (*M*_t_, *M*_r_, *M*_e_) = (4, 2, 2). Meanwhile, the secrecy outage probability with (*M*_t_, *M*_r_, *M*_e_) = (4, 2, 2) is higher than that with (*M*_t_, *M*_r_, *M*_e_) = (6, 3, 3). The same performance trend occurs when (*M*_t_, *M*_r_, *M*_e_) increases from (2, 1, 2) to (6, 3, 6) or from (2, 1, 3) to (6, 3, 9). These results reveal that adding *M*_t_ and *M*_r_ proportionally to *M*_e_ is advantageous.

**Figure 3:**
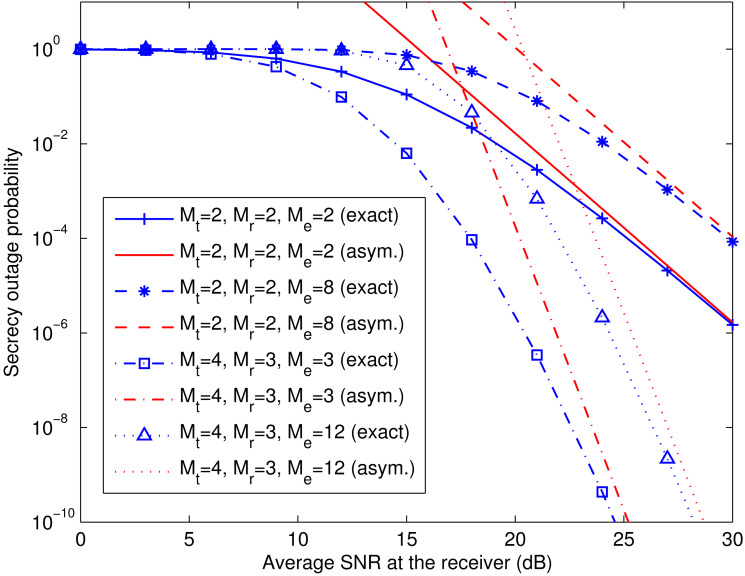
Comparison of exact and asymptotic secrecy outage probability of transmit-receive diversity system. This figure shows the exact and asymptotic secrecy outage curves for the transmit-receive diversity system with different numbers of antennas at the transmitter (*M*_t_), the legitimate receiver (*M*_r_), and the eavesdropper (*M*_e_). The exact and asymptotic results are labeled with “exact” and “asym.”, respectively.

Figure 3 verifies the asymptotic secrecy outage probability of the transmit-receive diversity system derived in Eqs. (24)–(26) at a fixed ${\bar {\gamma }}_{\text{e}}$ (i.e., ${\bar {\gamma }}_{\text{e}}=10$ dB). The exact and asymptotic secrecy outage curves are labeled with “exact” and “asym.”, respectively. As ${\bar {\gamma }}_{\text{r}}$ grows, the asymptotic curves approach the exact ones for different values of *M*_t_, *M*_r_, and *M*_e_. It can also be observed that the secrecy diversity gain is *M*_t_*M*_r_, as predicted by Eq. (25), and the secrecy array gain diminishes with increasing *M*_e_, as predicted by Eq. (26).

**Figure 4:**
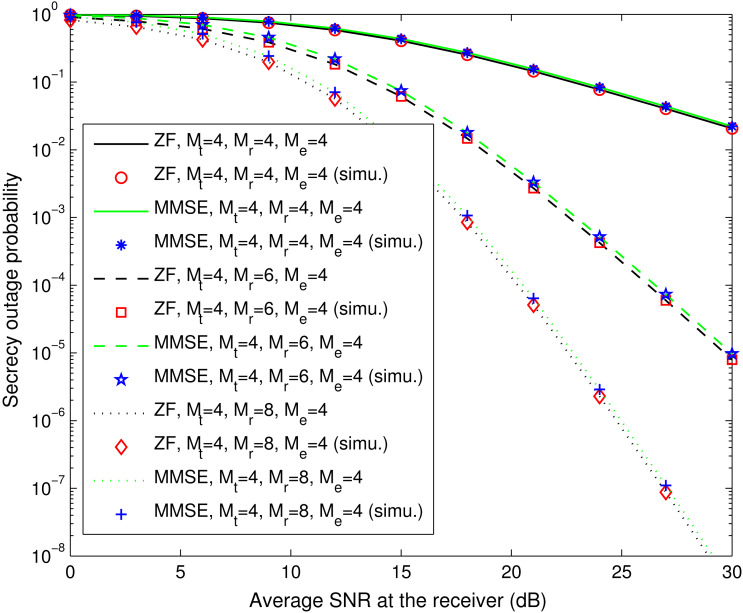
Secrecy outage probability of spatial multiplexing systems with ZF equalization (*P*_out,ZF_) and MMSE equalization (*P*_out,MMSE_). This figure shows the theoretical and simulated secrecy outage curves for the ZF equalization-based and MMSE equalization-based spatial multiplexing systems with different numbers of antennas at the legitimate receiver (*M*_r_) and fixed numbers of antennas at the transmitter (*M*_t_) and the eavesdropper (*M*_e_). The simulation results are labeled with “simu.”.

**Figure 5:**
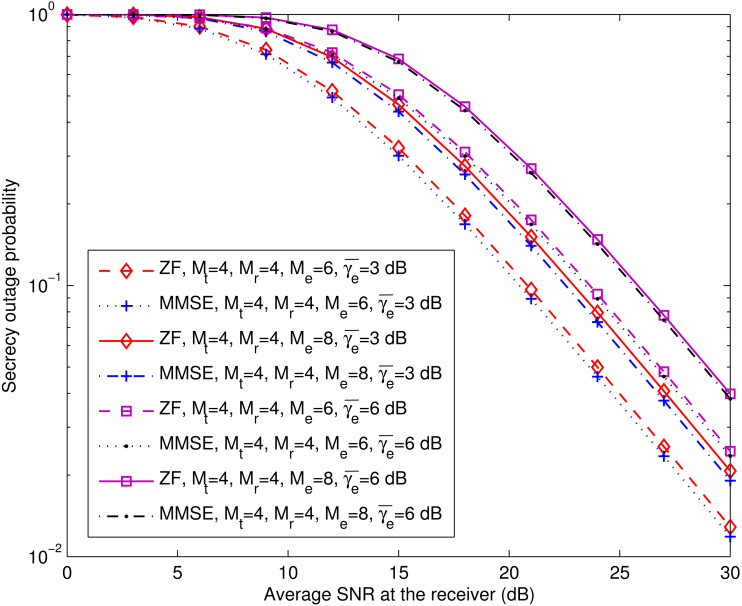
*P*_out,ZF_ versus *P*_out,MMSE_ for various *M*_e_ at fixed *M*_t_ and *M*_r_ (*M*_t_ = *M*_r_ = 4). This figure shows the theoretical secrecy outage curves for the ZF equalization-based and MMSE equalization-based spatial multiplexing systems with different numbers of antennas and average SNRs at the eavesdropper (*M*_e_ and ${\bar {\gamma }}_{\text{e}}$), and fixed numbers of antennas at the transmitter (*M*_t_) and the legitimate receiver (*M*_r_).

**Figure 6:**
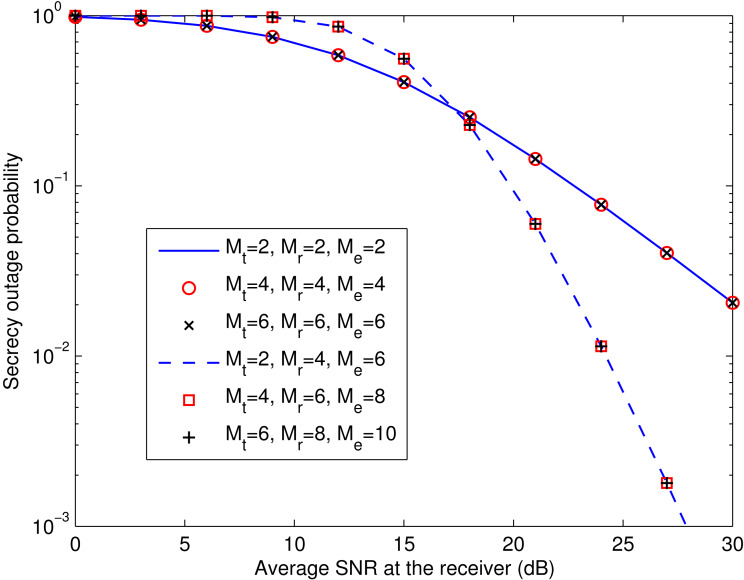
Examples of *P*_out,ZF_ with *M*_t_ = *M*_r_ = *M*_e_ and that with *M*_r_ = *M*_t_ + 2 and *M*_e_ = *M*_t_ + 4. This figure shows the simulated secrecy outage curves for the ZF equalization-based spatial multiplexing system in the case that the numbers of antennas at the transmitter (*M*_t_), the legitimate receiver (*M*_r_), and the eavesdropper (*M*_e_) are the same, and the case of *M*_r_ = *M*_t_ + 2, *M*_e_ = *M*_t_ + 4.

Figure 4 compares the theoretical secrecy outage results for the spatial multiplexing systems with ZF equalization (computed with Eq. (19)) and MMSE equalization (computed with Eq. (20)), and their simulation counterparts. The theoretical and simulation results agree well, and both kinds of systems exhibit similar secrecy outage performance. Indeed, the spatial multiplexing system with MMSE equalization achieves lower secrecy outage probability when the number of antennas at the eavesdropper is more than that at the receiver, as illustrated in Fig. 5. In addition, most noteworthy in Eq. (19) is the fact that, when the values of (*M*_r_ − *M*_t_) and (*M*_e_ − *M*_t_) are fixed, the secrecy outage probability of the spatial multiplexing system with ZF equalization remains the same regardless of the value of *M*_t_ that is used. This fact is confirmed by Fig. 6, where we plot the simulated secrecy outage curves in the case of *M*_r_ − *M*_t_ = 0, *M*_e_ − *M*_t_ = 0 and that of *M*_r_ − *M*_t_ = 2, *M*_e_ − *M*_t_ = 4.

**Figure 7:**
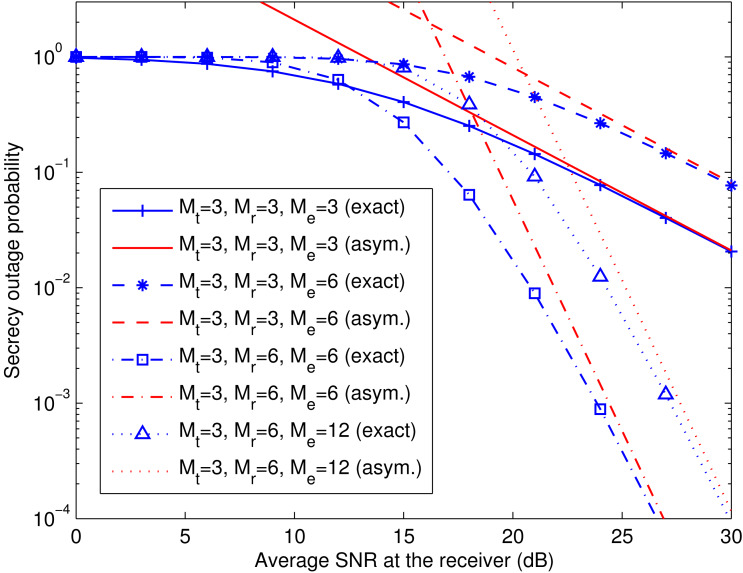
Comparison of exact and asymptotic secrecy outage probability of spatial multiplexing system with ZF equalization. This figure shows the exact and asymptotic secrecy outage curves for the ZF equalization-based spatial multiplexing system with different numbers of antennas at the legitimate receiver (*M*_r_) and the eavesdropper (*M*_e_), and a fixed number of antennas at the transmitter (*M*_t_). The exact and asymptotic results are labeled with “exact” and “asym.”, respectively.

**Figure 8:**
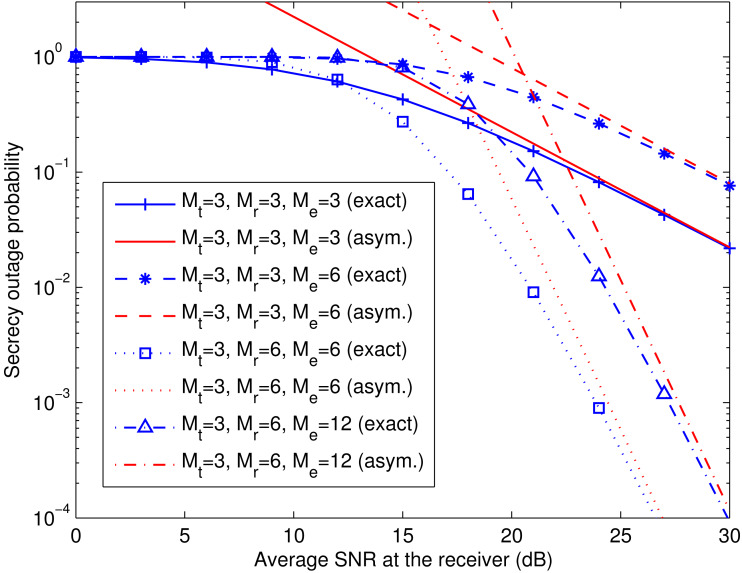
Comparison of exact and asymptotic secrecy outage probability of spatial multiplexing system with MMSE equalization. This figure shows the exact and asymptotic secrecy outage curves for the MMSE equalization-based spatial multiplexing system with different numbers of antennas at the legitimate receiver (*M*_r_) and the eavesdropper (*M*_e_), and a fixed number of antennas at the transmitter (*M*_t_). The exact and asymptotic results are labeled with “exact” and “asym.”, respectively.

Figures 7 and 8 verify the asymptotic secrecy outage probability of the spatial multiplexing system with ZF equalization derived in Eqs. (28)–(30) and that of the spatial multiplexing system with MMSE equalization derived in Eqs. (32)–(34), respectively, at a fixed ${\bar {\gamma }}_{\text{e}}$ (i.e., ${\bar {\gamma }}_{\text{e}}=10$ dB). As ${\bar {\gamma }}_{\text{r}}$ increases, the asymptotic curves tend towards the exact ones for different values of *M*_t_, *M*_r_, and *M*_e_. It can also be noticed that the secrecy diversity gains of the two systems are *M*_r_ − *M*_t_ + 1, as predicted by Eqs. (29) and (33), and the corresponding secrecy array gains lessen with growing *M*_e_, as predicted by Eqs. (30) and (34).

**Figure 9:**
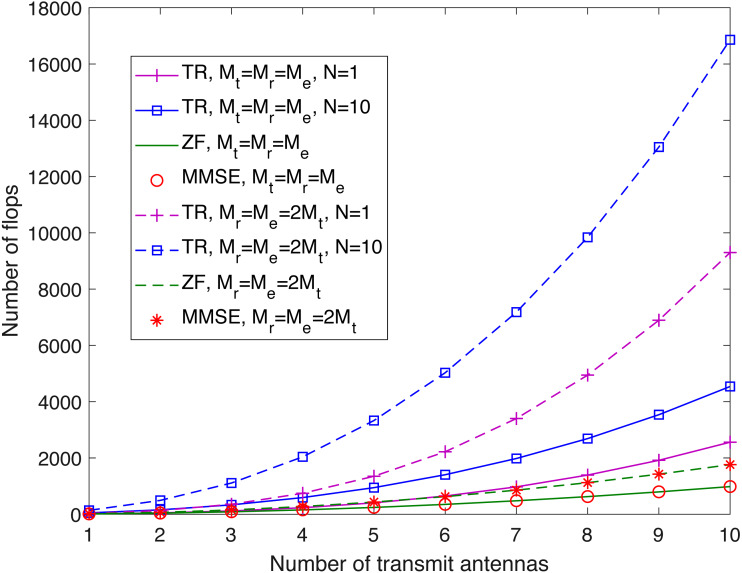
Comparison of system complexity for *M*_t_ = *M*_r_ = *M*_e_ and for *M*_r_ = *M*_e_ = 2*M*_t_. This figure shows the system complexity for the case that the numbers of antennas at the transmitter (*M*_t_), the legitimate receiver (*M*_r_), and the eavesdropper (*M*_e_) are the same, and the case of *M*_r_ = *M*_e_ = 2*M*_t_.

Finally, it is interesting to compare the computational complexity of all three systems. To this end, we express such complexity in terms of the number of floating-point operations (flops), and the relevant calculations are summarized as follows:^5^ 5For a detailed analysis of the number of flops required for matrix–vector operations such as associated summations and multiplications, readers are referred to Hunger (2007).(1) the number of flops required to compute **z**_r_ (via power iteration (Golub & Van Loan, 2013, Section 7.3)), **w**_t_, and **z**_e_ for the transmit-receive diversity system; (2) the number of flops required to compute **W**_r,ZF_ and **W**_e,ZF_ for the spatial multiplexing system with ZF equalization; and (3) the number of flops required to compute **W**_r,MMSE_ and **W**_e,MMSE_ for the spatial multiplexing system with MMSE equalization. The results are given in Table 1, where *N* is the number of iterations used in the power iteration method.^6^ 6In practice, the choice of *N* depends on the ratio between the magnitude of the second largest eigenvalue of ${\mathbf{H}}_{\text{r}}{\mathbf{H}}_{\text{r}}^{\dagger }$ and that of the corresponding largest eigenvalue as it dictates the rate of convergence (see Golub & Van Loan (2013), Section 7.3) for more details).Figure 9 shows the system complexity as a function of *M*_t_ for *M*_t_ = *M*_r_ = *M*_e_ and for *M*_r_ = *M*_e_ = 2*M*_t_. From this figure, we see that the computational complexity of the spatial multiplexing system with ZF equalization is comparable to that of the spatial multiplexing system with MMSE equalization, while the transmit-receive diversity system has the highest computational complexity, even with *N* = 1.

**Table 1:** System complexity in terms of floating-point operations. This table shows the computational complexity of the transmit-receive diversity system and the spatial multiplexing systems with ZF equalization and MMSE equalization.

System	Number of Flops
Transmit-Receive Diversity	$2{M}_{\text{t}}{M}_{\text{r}}^{2}+2{M}_{\text{t}}{M}_{\text{r}}+2{M}_{\text{t}}{M}_{\text{e}}+2{M}_{\text{t}}+(2N-1){M}_{\text{r}}^{2}+2N{M}_{\text{r}}+2{M}_{\text{e}}$
Spatial Multiplexing with ZF	$2{M}_{\text{t}}^{2}+4{M}_{\text{t}}{M}_{\text{r}}+4{M}_{\text{t}}{M}_{\text{e}}-{M}_{\text{r}}-{M}_{\text{e}}+2$
Spatial Multiplexing with MMSE	$2{M}_{\text{t}}^{2}+4{M}_{\text{t}}{M}_{\text{r}}+4{M}_{\text{t}}{M}_{\text{e}}-{M}_{\text{r}}-{M}_{\text{e}}+4$

## Conclusion

We have presented exact and asymptotic analysis of the secrecy outage probability of the transmit-receive diversity system and spatial multiplexing systems with ZF equalization and MMSE equalization in a Rayleigh-fading MIMO wiretap channel. This asymptotic analysis has shown that the transmit-receive diversity system achieves a secrecy diversity order of *M*_t_*M*_r_, whereas the two spatial multiplexing systems offer the same secrecy diversity order of *M*_r_ − *M*_t_ + 1. Interestingly, all of these secrecy diversity orders do not rely on *M*_e_. Numerical results based on both theoretical analysis and simulations have demonstrated how *M*_t_, *M*_r_, and *M*_e_ affect the secrecy performance of such MIMO systems.

## Additional Information and Declarations

### Data Availability

The following information was supplied regarding data availability: Data is available at GitHub: https://github.com/Secrecy1234/peerj.
